# Positive predictive value of Interferon-gamma release assay for incident active tuberculosis in HIV-infected persons

**DOI:** 10.1186/1742-4690-9-S1-P133

**Published:** 2012-05-25

**Authors:** Susan Shin-Jung Lee, Hsi-Hsun Lin, Hung-Chin Tsai, Yen-Yun Ni, Yao-Shen Chen, Chi-Tai Fang

**Affiliations:** 1 Kaohsiung Veterans General Hospital, Kaohsiung, Taiwan, Province of China

## Introduction

Tuberculosis (TB) is the leading cause of death in HIV/AIDS. The incidence of active TB is 30 times higher in HIV-infected persons. Treatment of latent TB infection (LTBI) is pivotal to the control of TB. We aim to determine the positive predictive value of an interferon-gamma release assay, QuantiFERON-TB GOLD test (QFT), for incident active TB in HIV-infected persons.

## Materials and methods

This prospective, 5-year, cohort study enrolled HIV-infected adults without active TB. Demographic data, past exposure to TB and previous TB disease, HIV risk factors, CD4 counts, and HIV viral loads were recorded. QFT tests were done at entry. Cases of incident TB disease were ascertained by linking to our national TB database registry.

## Results

We recruited 774 HIV-infected adults, with a mean age of 36.9 years, mostly men (96.8%). HIV risk factors included intravenous drug user (67.2%), men-who-have-sex-with-men (24.2%), and heterosexual (7.9%). QFT was positive in 90 (11.6%,95% CI: 9.5-14.1%) and indeterminate in 31 (4.0%). On multivariate logistic regression analysis, significant risk factors for QFT positivity included older age, females, past TB disease and exposure to TB.

Fifteen incident active TB cases (rate: 5.19/1000 person-years 95%CI:3.13-8.61) occurred during a mean follow up time of 3.73 person-years, with the majority (91.3%) followed up for over 2 years. Incident active TB disease occurred in 5.6% (5/90) of those with a positive QFT result, 3.2% (1/31) indeterminate results, and 1.4% with a negative QFT result (p=0.03). Hazard ratio for developing active TB was 3.10 (95%CI:1.03-9.30, p=0.04) for a positive QFT and 1.72 (p=0.60) for an indeterminate QFT result.

## Conclusions

Our study demonstrated that QFT test predicted incident active TB disease in HIV-infected persons, with a hazard ratio of 3.10. QFT can be used for diagnosis of LTBI in HIV-infected persons, to allow targeted treatment in this high risk group.

